# A Case of a Large and Rare Incidental Pleural Tumor in an Elderly Female

**DOI:** 10.7759/cureus.42198

**Published:** 2023-07-20

**Authors:** Ruchi Mangal, Muneer F Hasso, Mark S Obri, Mohamed Ramzi Almajed, Abigail Entz

**Affiliations:** 1 Internal Medicine, Henry Ford Health System, Detroit, USA

**Keywords:** cancerous mass effect, rare malignancies, incidental radiological finding, pleural tumor, solitary fibrous tumor (sft)

## Abstract

Solitary fibrous tumors are very rare in the pleura, and they are generally found incidentally. Even though they can potentially become malignant and metastasize, they have minimal clinical symptoms and can still be benign. Due to the low incidence of these tumors, there is no standard of therapy beyond surgical resection. We present an asymptomatic case of a large, rapidly expanding solitary fibrous tumor of the pleura in an elderly female.

## Introduction

A solitary fibrous tumor of the pleura (SFTP) is rare and generally found incidentally; it accounts for <5% of all pleural tumors [1]. SFTP is a mesenchymal neoplasm, which generally involves the pleura, but can also be found in other thoracic areas such as the mediastinum, pericardium, and lung. There are also extrathoracic findings of SFTPs seen in the epiglottis, salivary glands, thyroid, kidneys, and breast. They often present as benign, but they have the potential to progress to malignancy even with minimal clinical symptoms [2]. The few solitary fibrous tumors with malignant potential show locally invasive properties or relapse after surgical resection. Due to their rarity, there is no standard of therapy for these tumors beyond resection. A diagnosis can be made through pre-operative core needle biopsy but has distinctive features seen on imaging [3]. We present a case of a large, rapidly expanding, yet asymptomatic SFTP.

This article was previously presented as a meeting abstract at the 2023 MI-ACP Resident and Student Day on May 12, 2023.

## Case presentation

A 76-year-old female, who is known hypertensive and has a history of >80 pack years of smoking history, osteoarthritis, and numerous falls, presented to the ED for falls and right-sided body pain. She was unable to provide information due to her altered mental status. Her daughter endorsed that her cognition had declined from a baseline of being alert and independent in the past 30 days with an unclear inciting factor.

**Figure 1:**
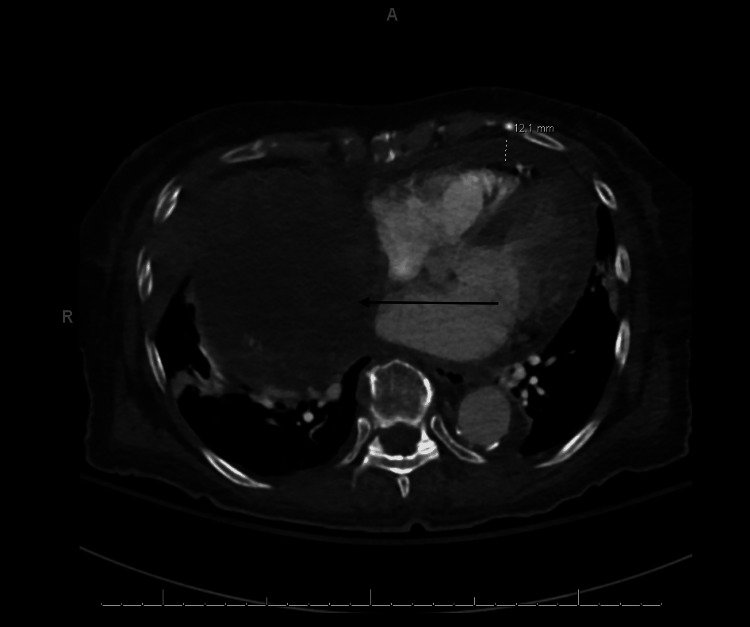
CT pulmonary abdomen pelvis (axial view). This figure shows an axial view of a CT scan of the pulmonary abdomen pelvis, highlighting the superior vena cava (SVC) and right atrium being compressed by a large ovoid, solid-appearing, heterogeneously enhancing mass (indicated by the arrow).

**Figure 2:**
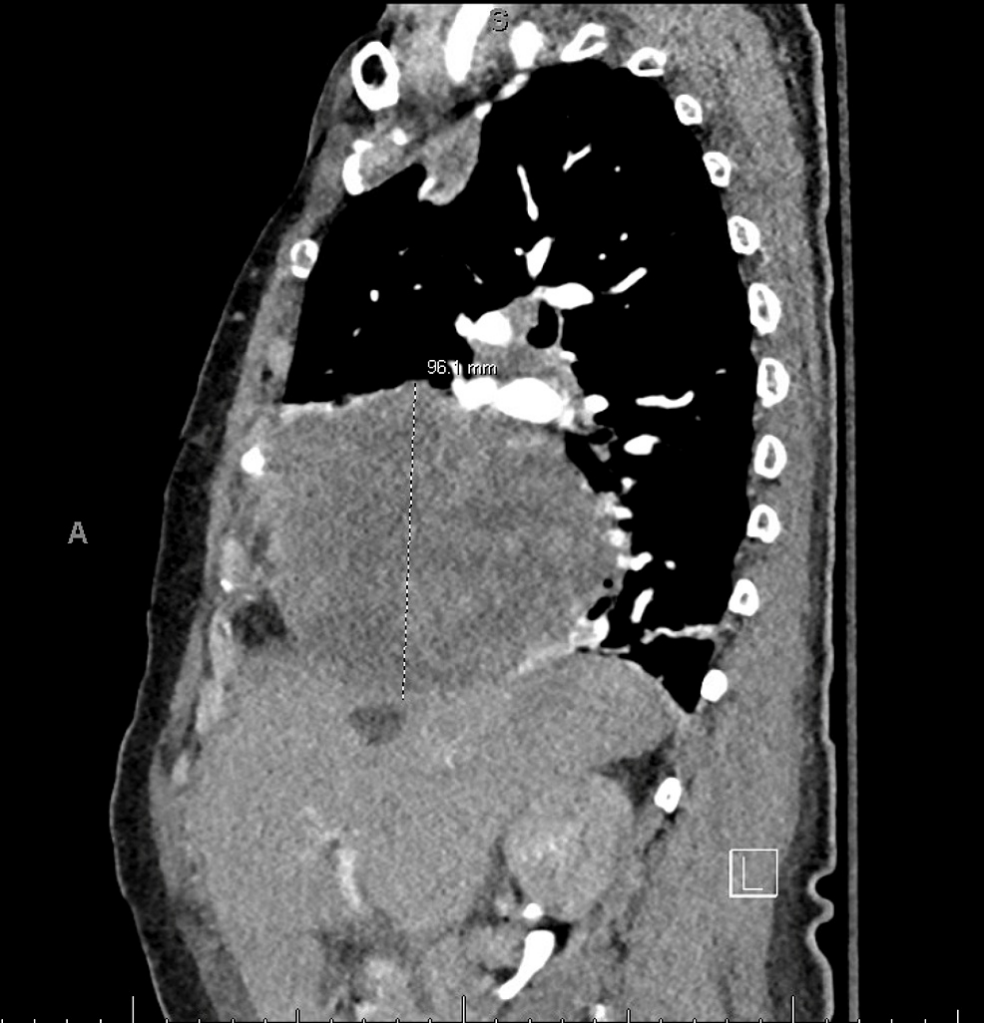
CT pulmonary abdomen pelvis (sagittal view). This figure shows a sagittal view of a CT pulmonary abdomen pelvis, showing a large mass occupying the anterior inferior half of the hemithorax that is approximately 12 cm traverse by 10 cm AP by 10 cm CC.

On initial presentation, she was found to be hypertensive and ill-appearing with regular respiratory effort. Her physical exam was significant only for lower extremity edema and weakness, contributing to her falls. Her initial labs in the ED were significant for a hemoglobin of 7.8 g/dl (baseline of 12.5 g/dl in 2018), and her D-dimer was elevated to 3.49 ug/mL fibrinogen equivalent units. Computed tomography (CT) of the head showed bilateral subdural hematomas with mild mass effect and no midline shift. A CT pulmonary abdomen pelvis was ordered due to the elevated D-dimer and was found to be negative for pulmonary embolism but revealed a 12 cm heterogeneously enhancing solid mass throughout the anterior inferior right hemothorax, suspicious for an SFTP. The mass encased the right middle lobe bronchovascular structures and moderately compressed the superior vena cava/right atrium, mildly displacing the heart slightly to the left, with no invasion of the chest wall or mediastinum (Figures 1-2).

**Figure 3:**
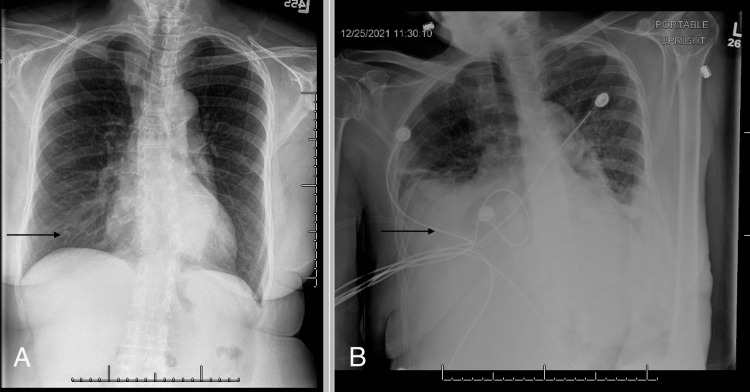
Comparison of chest X-ray between 2017 and 2021. This figure compares a chest radiograph in 2017 (A) to one in 2021 (B). The prior X-ray (A) was negative for pathological processes. The newer X-ray (B) shows a pleural effusion consistent with a large mass (see arrow).

The patient had a chest radiograph (CXR) done in 2017 that showed no acute process (Figure 3). A subsequent radiograph was taken in 2021 for comparison.

A biopsy was not done due to the risks associated with the procedure, including airway compression and bleeding. It was concluded that this mass was likely a solitary fibrous tumor due to its rapid onset, morphologic presentation, and potential for malignancy. The patient began declining rapidly through respiratory distress and hypoxia, thought to be due to a likely pulmonary embolism in the setting of malignancy. She continued to become more hypoxic, and the decision was made to pursue comfort care.

## Discussion

We present a rare case of a solitary fibrous tumor. Although it is typically benign, it holds significant malignant potential. Remarkably, it often presents either asymptomatically or with minimal pulmonary symptoms [4,5]. Around 50% of solitary fibrous tumors are discovered incidentally during chest imaging procedures like CXR or CT [6]. Despite their typically minimal symptoms, these tumors can invade cardiac tissue, leading to embolization and contributing to other critical conditions. The insidious ability of these tumors to grow rapidly and asymptomatically can have severe consequences for patients [7]. They are prone to causing symptoms of mass effect. In our case, the patient's tumor had grown to 12 cm, compressing the superior vena cava and right atrium, which may have contributed to the patient's acute decompensation and hypoxia. Although a biopsy was not performed in this case to confirm the diagnosis, the imaging presentation of the tumor was consistent with previous cases where a biopsy confirmed the diagnosis of SFTP [3]. The rapid onset and morphological presentation further reinforced suspicions of an SFTP in this case. The current mainstay therapy for these tumors is resection [8,9]. However, due to our patient's comorbidities and acute decompensation, surgery was not a viable option. While impressive, solitary pulmonary tumors may not always have clinical relevance. Future studies should focus on the necessity of resecting asymptomatic SFTP. Additionally, further investigation into the disease progression and growth rates of SFTP is warranted [10,11].

## Conclusions

SFTPs are rare and often found incidentally. They generally do not present with clinical symptoms despite their size, as seen in this case. Currently, the reported cases categorize these tumors as unresectable or treated with surgery only. It would be beneficial to do additional future studies on the benefits of targeted therapies in patients that are not surgical candidates. These tumors can still have significant malignant potential and can adversely affect nearby structures, warranting further investigation.
